# Unveiling the Mitochondrial Mystery: A Case of Mitochondrial Encephalopathy With Lactic Acidosis and Stroke-Like Episodes

**DOI:** 10.7759/cureus.72397

**Published:** 2024-10-25

**Authors:** Nipuna Weerasinghe, Madhawa Weerasinghe, Kishan Dissanayake, Hemal Hordagoda, Janaka Peiris

**Affiliations:** 1 Neurology, National Hospital Kandy, Kandy, LKA; 2 Radiology, National Hospital Kandy, Kandy, LKA

**Keywords:** epilepsy, gyriform restricted diffusion, magnetic resonance spectroscopy, mitochondrial encephalopathy, retinitis pigmentosa

## Abstract

A 38-year-old female presented with difficulty walking and focal seizures causing non-specific facial and upper limb movements. Examination revealed nystagmus and other peripheral cerebellar signs. Lower limbs were spastic with up-going plantar responses bilaterally. She had been on treatment for epilepsy with multiple anti-seizure medications since the age of 20 and had an episode of status epilepticus when she was 25 years old. An MRI scan of the brain revealed focal gyriform restricted diffusion, and she was followed up with an MR spectroscopy, which revealed lactate peaks involving the affected area as well as the ventricular system in the brain. Her cerebrospinal fluid lactate levels were also elevated. She was also found to have retinitis pigmentosa on dilated fundoscopy. In the absence of genetic studies, due to financial restrictions, she was diagnosed with mitochondrial encephalopathy with lactic acidosis and stroke-like episodes (MELAS) based on the overall clinical picture. Sodium valproate was discontinued as it had toxic properties against mitochondria, and she was put on co-enzyme Q10, L-arginine, and carnitine. This case highlights the difficulties faced in diagnosing conditions like MELAS in a setting where genetic studies are not feasible.

## Introduction

Neurological disorders with mitochondrial inheritance are a group of rare diagnoses that require a high index of suspicion. Among these, mitochondrial encephalopathy with lactic acidosis and stroke-like episodes (MELAS) is rare, and a limited number of cases have been reported from Sri Lanka. Mitochondrial disorders as a whole are under-reported, especially due to the limited availability of genetic studies [1]. Therefore, it is important to find available surrogate investigations to confirm the diagnosis of MELAS in a resource-poor setting.

A 38-year-old girl who was under long-term follow-up for epilepsy presented with difficulty walking. When the diagnosis was re-visited, there were pointers towards MELAS. Therefore, she underwent further confirmatory investigations, which helped establish the diagnosis. This case highlights the importance of revisiting an already established diagnosis and the relevance of brain imaging in diagnosing MELAS. This case was presented as an abstract at the Association of Sri Lankan Neurologists Annual Congress (ASNAC) 2024.

## Case presentation

A 38-year-old female who was living with her mother presented with intermittent focal-onset seizures with preserved awareness going on for two days. She was on treatment for seizures with good compliance for almost 20 years. She was also complaining of difficulty walking due to ataxia. Neurological examination revealed nystagmus, with finger-nose test positivity on the right side and a broad-based gait. She had brisk reflexes with up-going plantar bilaterally. Her cognitive functions were satisfactory, with a mini-mental score of 24. While going through her past records, it was noted that in 2010, she had been intubated and ventilated at an intensive care unit for status epilepticus, at which point she had undergone several MRI scans of the brain, all of which had revealed a large right parieto-occipital infarct. She had been on antiplatelet therapy since then, and she had been compliant with the medication. She also had undergone surgery for cataracts involving both eyes when she presented with gradual visual impairment two years prior to the current presentation. She was not carrying significant risk factors for atherosclerotic cardiovascular disease such as diabetes mellitus or hypertension. There was no family history suggestive of a heritable neurological condition.

**Figure 1:**
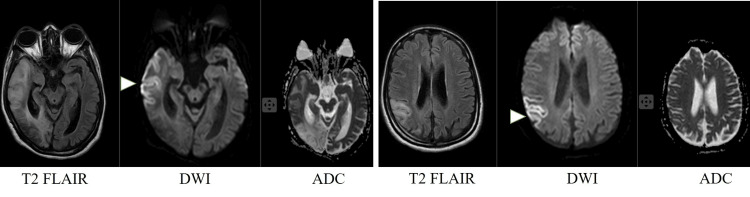
MRI scan of the brain revealing GRD Arrowheads reveal areas of GRD as hyperintensities on DWI with corresponding hypointensities in ADC images. GRD: Gyriform restricted diffusion, DWI: Diffusion-weighted imaging, ADC: Apparent diffusion coefficient

**Figure 2:**
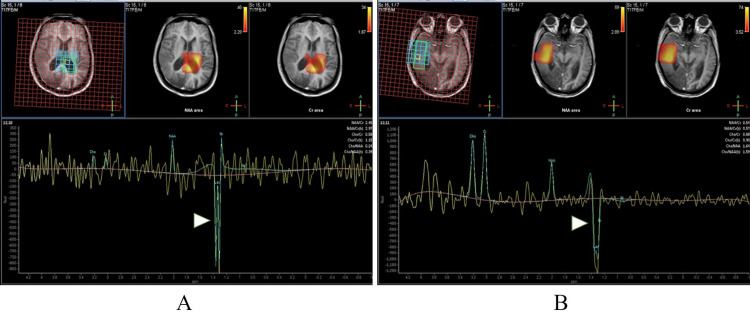
MR spectroscopy of the brain Arrowheads denote lactate peaks involving the lateral ventricles (A) and brain parenchyma (B).

Basic blood workup did not reveal significant abnormalities except for a slightly elevated blood lactate level (3.0 mmol/l). Her full blood count revealed a hemoglobin level of 10.5 g/dl and a platelet count of 330 000/microliter. Erythrocyte sedimentation rate (ESR) and C-reactive protein (CRP) levels were within the normal range. The blood picture did not reveal any abnormalities, and her clotting profile was normal. There were no investigational findings suggestive of an underlying thrombophilic condition. Cardiac workup did not reveal a clear source for recurrent embolism in the brain. The MRI scan of the brain revealed extensive gyriform restricted diffusion (GRD) involving the right parieto-occipital lobes (Figure 1). Since MELAS was considered a possible diagnosis, an MR spectroscopy of the brain was arranged, which revealed lactate peaks involving the brain parenchyma and the ventricles (Figure 2). Her cerebrospinal fluid lactate levels were also raised.

To gather further evidence, the patient underwent a muscle biopsy (deltoid muscle), which did not reveal the presence of red-ragged fibers. She was referred to the ophthalmologist for dilated ophthalmoscopy. There was evidence of retinal involvement of a mitochondrial disorder with the presence of retinitis pigmentosa. Unfortunately, the family was not able to afford genetic studies to find out the exact mitochondrial genetic mutation responsible for MELAS in this patient.

For the management of MELAS, the patient started immunonutrition therapy. She was prescribed co-enzyme Q10, L-arginine, and carnitine. However, due to the limited availability of these medications in the public health system, she was only able to afford some of them, and that too not continuously. Antiplatelet and statin therapy was discontinued since there was no evidence for these medications in the context of MELAS. More importantly, sodium valproate was changed to oxcarbazepine due to the former drug’s mitochondrial toxic properties. Seizure control was achieved with this change of medications, and she continued to function independently.

## Discussion

Mitochondrial encephalopathy with lactic acidosis and stroke-like episodes is a rare, maternally-inherited disorder and is one of a long list of neurodegenerative disorders that occur as a result of mitochondrial dysfunction. Mutations in either mitochondrial or nuclear DNA result in defective mitochondrial protein synthesis, which leads to disruption of aerobic metabolism and cellular energy production. This in turn leads to various clinical manifestations, most of which are neurological or muscle-related [2]. Therefore, clinical presentation could mimic many other neurological disorders, and it is vital to have a high degree of suspicion to promptly diagnose MELAS. Moreover, several genetic characteristics also contribute to the heterogeneous clinical presentation of these mitochondrial disorders.

Mitochondrial encephalopathy with lactic acidosis and stroke-like episodes is most commonly secondary to a transfer RNA (tRNA) variation caused by a mutation in the MTTL1 mitochondrial gene. When it comes to mitochondrial genetic mutations, mitochondria are randomly assigned during mitosis; each cell will have a varying amount of mutant mitochondrial DNA. This phenomenon is referred to as heteroplasmy. Once the amount of mutant DNA surpasses a threshold amount, the mitochondrial function and, in turn, cellular function get disrupted. This mainly affects tissues with a high energy requirement, such as neural tissue and skeletal muscles [3]. The genetic bottleneck effect is also postulated to play a major role in the genetics of mitochondrial disorders. Accordingly, only a small amount of maternal mitochondrial DNA gets transmitted to the progeny, contributing to a varying heteroplasmy among members in a given generation. Succinctly, concerning maternally inherited mitochondrial disorders, different members of the same generation will get affected to different degrees with different organ dysfunctions, and different tissues in a given member will have a varying amount of the mutant mitochondrial DNA.

Diagnosing MELAS in a setting where genetic studies are not freely available is a challenge. It requires gathering evidence using various other investigations such as serum lactate level, cerebrospinal fluid lactate level, brain imaging, muscle biopsy, screening for retinal involvement of mitochondrial disorders, etc. As genetic studies were not feasible for our patient, brain imaging played a major role in diagnosing MELAS. Diffusion-weighted imaging (DWI) and apparent diffusion coefficient (ADC) are two MRI sequences that are commonly used in the assessment of acute ischaemic strokes. Due to the ischaemic insult, there is dysfunction of the sodium/potassium pumps on the cell membrane. This, in turn, leads to the accumulation of fluid within the cell, causing cytotoxic edema. The lack of transmembrane motion of water, which is a 'restriction' of the Brownian motion of water molecules, causes a high signal in the DWI and a low signal in the ADC images. As this ischaemic insult affects gray matter where neuronal cell bodies are located, diffusion restriction is seen along the gyri. Therefore, these changes are termed GRD [4].

Although ischaemic stroke is the most commonly recognized cause of GRD, there are a few other causes that need to be considered when GRD is seen on an MRI. Mitochondrial encephalopathy with lactic acidosis and stroke-like episodes is among these causes; the other causes are hypoxic ischaemic encephalopathy, hypoglycemia, hyperammonaemia, herpes encephalitis, post-ictal changes, and Creutzfeldt-Jakob disease (CJD) [4]. In MELAS, as mitochondrial dysfunction occurs, the previously mentioned adenosine triphosphate (ATP)-dependent sodium/potassium channels are affected due to the lack of efficient production of ATP. However, since cellular involvement could be patchy in mitochondrial disorders, GRD is not uniformly seen throughout the brain. Therefore, in the appropriate clinical setting, GRD can be utilized as a surrogate investigation to diagnose MELAS.

In MELAS, GRD has a predilection to the temporo-parietal and occipital lobes, as in our patient. The fact that GRD in MELAS does not conform to a particular vascular territory helps differentiate it from ischaemic strokes. Lesions are also known to show a waxing and waning pattern, which also makes them distinguishable from other causes of GRD [4]. Additionally, MR spectroscopy is useful in further confirming a diagnosis of MELAS. With regard to our patient, a lactate peak was evident on MRS at 1.33 ppm. This further supports the diagnosis of MELAS in her. The lactate production is secondary to the anaerobic metabolism, which takes place within this affected area. The produced lactate can often leak into the subarachnoid space, resulting in a rise in the cerebrospinal fluid lactate level. Therefore, as in our patient, a rise in cerebrospinal fluid lactate further confirms the diagnosis of MELAS.

Our patient was initially managed for epilepsy secondary to a past ischaemic injury. She was on long-term aspirin and three anti-seizure medications, including sodium valproate. Following the diagnosis of MELAS, aspirin was discontinued as there is no evidence for the use of antiplatelets to prevent future stroke-like episodes [5]. As for anti-seizure medications, sodium valproate is contraindicated in mitochondrial epilepsy due to its toxic effects on mitochondria [5]. Therefore, it is possible that she continued to get seizures despite good compliance due to the continued administration of sodium valproate. Once she was put on alternative anti-seizure medications, her seizures disappeared.

There is no curative therapy for MELAS, and treatment focuses on the administration of nutrient-rich diets that modulate the immune system. Both L-arginine and citrulline, per the literature, have been used in the management of stroke-like episodes [2]. The postulated mechanism of action of these nitric oxide precursors is the replenishment of nitric oxide, helping with cerebral vasodilation. There are reported instances where L-arginine is administered intravenously as infusions during an acute attack or a stroke-like episode. Other treatment options include co-enzyme Q10, vitamin K1, vitamin K3, and ascorbate, which provide electrons to cytochrome c [2]. Idebenone, which is a synthetic co-enzyme Q10, is undergoing phase II trials and showing promise in reducing neurological disabilities in patients with MELAS [6].

## Conclusions

The case we have reported offers valuable insights into diagnosing MELAS in adults. One crucial takeaway is the necessity of revisiting initial diagnoses, even when they seem definitive. Additionally, challenges encountered in diagnosing MELAS without genetic testing highlight the importance of thorough evaluation and exploration of various diagnostic tools. Furthermore, the case serves as a stark reminder of the potential adverse effects of valproate in patients with MELAS, emphasizing the need for careful medication selection in such cases. By understanding these lessons, healthcare providers can improve their diagnostic accuracy and treatment outcomes for patients with MELAS.

## References

[1] Hettiarachchi D, Lakmal K, Dissanayake VH (2022). Mitochondrial diseases in South Asia — a systematic review. Mitochondrion.

[2] Pia S, Lui F (2024). Melas Syndrome. Treasure Island.

[3] Burr SP, Chinnery PF (2024). Origins of tissue and cell-type specificity in mitochondrial DNA (mtDNA) disease. Hum Mol Genet.

[4] Pai V, Sitoh YY, Purohit B (2020). Gyriform restricted diffusion in adults: looking beyond thrombo-occlusions. Insights Imaging.

[5] Ng YS, Bindoff LA, Gorman GS (2019). Consensus-based statements for the management of mitochondrial stroke-like episodes. Wellcome Open Res.

[6] Montenegro L, Turnaturi R, Parenti C, Pasquinucci L (2018). Idebenone: novel strategies to improve its systemic and local efficacy. Nanomaterials (Basel).

